# Chest CT features associated with the clinical characteristics of patients with COVID-19 pneumonia

**DOI:** 10.1080/07853890.2020.1851044

**Published:** 2021-01-10

**Authors:** Ruichao Niu, Shuming Ye, Yongfeng Li, Hua Ma, Xiaoting Xie, Shilian Hu, Xiaoming Huang, Yangshu Ou, Jie Chen

**Affiliations:** a Department of Respiratory Medicine, Xiangya Hospital, Central South University, Changsha, PR China; b Department of Respiratory Medicine, Wuhan First Hospital/Wuhan Hospital of Traditional Chinese and Western Medicine, Wuhan, PR China; c Department of Respiratory Medicine, Anyang District Hospital, Anyang, PR China; d Department of Infectious Disease, People’s Hospital of Liuyang City, Liuyang, PR China; e Department of Respiratory Medicine, People’s Hospital of Ningxiang City, Ningxiang, PR China; f Department of Radiology and Imaging, The Third Hospital of Yongzhou City, Yongzhou, PR China; g Department of Radiology and Imaging, Traditional Chinese Medicine Hospital of Leiyang City, Hengyang, PR China

**Keywords:** COVID-19, pneumonia, SARS-CoV-2 imaging features, computed tomography

## Abstract

**Objectives:**

Coronavirus disease 2019 (COVID-19) has rapidly swept across the world. This study aimed to explore the relationship between the chest CT findings and clinical characteristics of COVID-19 patients.

**Methods:**

Patients with COVID-19 confirmed by next-generation sequencing or RT-PCR who had undergone more than 4 serial chest CT procedures were retrospectively enrolled.

**Results:**

This study included 361 patients – 192 men and 169 women. On initial chest CT, more lesions were identified as multiple bilateral lungs lesions and localised in the peripheral lung. The predominant patterns of abnormality were ground-glass opacities (GGO) (28.5%), consolidation (13.0%), nodule (23.0%), fibrous stripes (5.3%) and mixed (30.2%). Severe cases were more common in patients with a mixed pattern (21.1%) and less common in patients with nodules (2.4%). During follow-up CT, the mediumtotal severity score (TSS) in patients with nodules and fibrous strips was significantly lower than that in patients with mixed patterns in all three stages (*p* < .01).

**Conclusion:**

Chest CT plays an important role in diagnosing COVID-19. The CT features may vary by age. Different CT features are not only associated with clinical manifestation but also patient prognosis.

**Key messages**
1. The initial chest CT findings of COVID-19 could help us monitor and predict the outcome.
2. Nodules were more common in non severe cases and had a favorable prognosis.
3. The mixed pattern was more common in severe cases and usually had a relatively poor outcome.

## Introduction

Coronavirus disease 2019 (COVID-19), caused by severe acute respiratory syndrome coronavirus 2 (SARS-CoV-2), was first reported in Wuhan, a city in China’s Hubei province. Because of the strong affinity of SARS-CoV-2 for human respiratory epithelial cells, COVID-19 is highly infectious and rapidly spread from Wuhan to the entire country and overseas. By 10 August 2020, 19,915,054 laboratory-confirmed cases of COVID-19 pneumonia with 728,500 deaths had been reported in China and in other countries worldwide (including Thailand, Japan, South Korea, and the USA). COVID-19 poses a serious threat to human health and safety. Therefore, the World Health Organization listed COVID-19 as a public health emergency of international concern.

COVID-19 is diagnosed mainly by reverse transcription-polymerase chain reaction (RT-PCR) to detect SARS-CoV-2 nucleic acid. However, due to improper clinical sampling, low patient viral load and variation in the detection rate of different RT-PCR kits, the sensitivity of RT-PCR for COVID-19 infection is only 71% [1]. Additionally, chest radiographs are less sensitive than chest CT, particularly in the early stage of COVID-19 [2]. According to current reports, CT may be able to detect the disease prior to the development of clinical symptoms [3,4]. Therefore, chest CT is vital in preclinical screening and strongly recommended as a first-line strategy for investigating possible COVID-19 cases [5].

With growing global concerns about the COVID-19 epidemic, numerous studies have reported the CT features of COVID-19 pneumonia. However, specific data regarding how CT features are related to clinical characteristics are scarce. Additionally, the evolution of chest CT findings and their relation to the disease progression of COVID-19 has not been fully elucidated.

Herein, we investigated the epidemiological, clinical, laboratory test and chest CT findings of 361 patients with laboratory-confirmed COVID-19. We also aimed to define the evolution of CT findings associated with the clinical symptoms, laboratory testing, and disease progression of COVID-19.

## Methods

### Study design and patients

This retrospective study was approved by the Institutional Review Board of Wuhan First Hospital (2,020,032 K). The requirement for informed patient consent was waived by the institutional ethics committee because they were no risks associated with this study and would not adversely affect the subjects’ rights or welfare. COVID-19 was diagnosed based on the World Health Organisation (WHO) interim guidance and was eventually confirmed by a positive result from high-throughput sequencing or real-time reverse transcription polymerase chain reaction (RT-PCR) analysis of respiratory secretion samples [6,7].

All consecutive patients with COVID-19 pneumonia who were admitted to Wuhan First Hospital from 4 January 2020 to 20 March 2020 were included. The inclusion criteria were patients who had undergone initial chest CT less than 5 days from illness onset, had undergone serial chest CT (once every 4–5 days for more than 4 times) and had not received any antiviral treatment. The exclusion criteria were patients with insufficient quality of chest CT images for analysis. All eligible patients were followed up until 30 April 2020. Their demographic data, personal history, exposure history, underlying comorbidities, signs and symptoms, laboratory findings, treatment measures, and outcomes were collected by two experienced respiratory medical doctors from electronic medical records.

### Chest CT image acquisition

All CT examinations were performed using two commercial multi detector CT scanners (SOMATOM Definition AS; Siemens Healthineers, Germany; Bright Speed, GE Healthcare, America) without using contrast material. All the patients were scanned in the supine position and at the suspended end-inspiratory volume. The scanning range was from the thoracic inlet to the costophrenic angle. The scan parameter settings were as follows: tube voltage of 120 kV (automatic tube current modulation) and detector collimation widths of 128 × 0.6 mm. Primary image reconstruction was performed using a matrix size of 512 × 512 as axial images, 1.0-mm slice thickness,1mm increments, and a sharp reconstruction kernel.

### Chest CT evaluation

All chest CT scans were independently reviewed using the institutional digital database system (Vue PACS, version 11.3.5.8902, Care stream Health, Canada) by two radiologists (X.X. and H.S.) with 5 and 3 years of experience in imaging, respectively, and final decisions were reached by consensus. All CT images were described according to the Fleischner Society guide lines and peer-reviewed literature on viral pneumonia. The following CT features were evaluated: appearance (ground-glass opacities [GGO], consolidation, nodule, fibrous stripes); distribution (peripheral, central, or central and peripheral), number of lobes involved, specific signs of foci (vascular thickening, air bronchogram sign, halo sign, and fibrosis), and extra pulmonary manifestations (mediastinal and hilar lymph node enlargement, pleural effusion). A semi quantitative score was assigned for each of the five lung lobes: none (<5%), minimal (5–25%), mild (26–49%), moderate (50–75%), or severe (76–100%), with corresponding scores of 1, 2, 3, 4, or 5, respectively. The total severity score (TSS) was the sum of the individual lobar scores (ranging from 1 to 25) [8].

The findings were divided into three stages according to follow-up chest CT scans. The evolution of lesions was assessed by comparing the scope, quantity, and density of lesions between the two adjacent chest CT examinations from the same patient. Progression was defined as the appearance of new lesions and/or an increase in lung density and/or enlargement of the lesions. Conversely, absorption was defined as a decrease in lung density and/or reduction in the scope or number of lesions. Stable was defined as no significant difference in the lesions between the two CT examinations.

### Statistical analysis

Analyses were performed using the Statistical Package for the Social Sciences software version 20.0 (Chicago, IL, USA). Continuous variables were expressed as either means and standard deviation (SD) or medians (interquartile range, IQR), and comparison between groups was performed using *t-*test, the Mann–Whitney *U* test, and Kruskal–Wallis test when appropriate. Categorical variables were expressed as numbers (percentages), and comparisons between groups were performed by chi-square test and Fisher’s exact test. Statistical significance was defined as a *p* <.05.

## Results

### Demographic and epidemiologic characteristics and clinical symptoms

**Figure 1.**
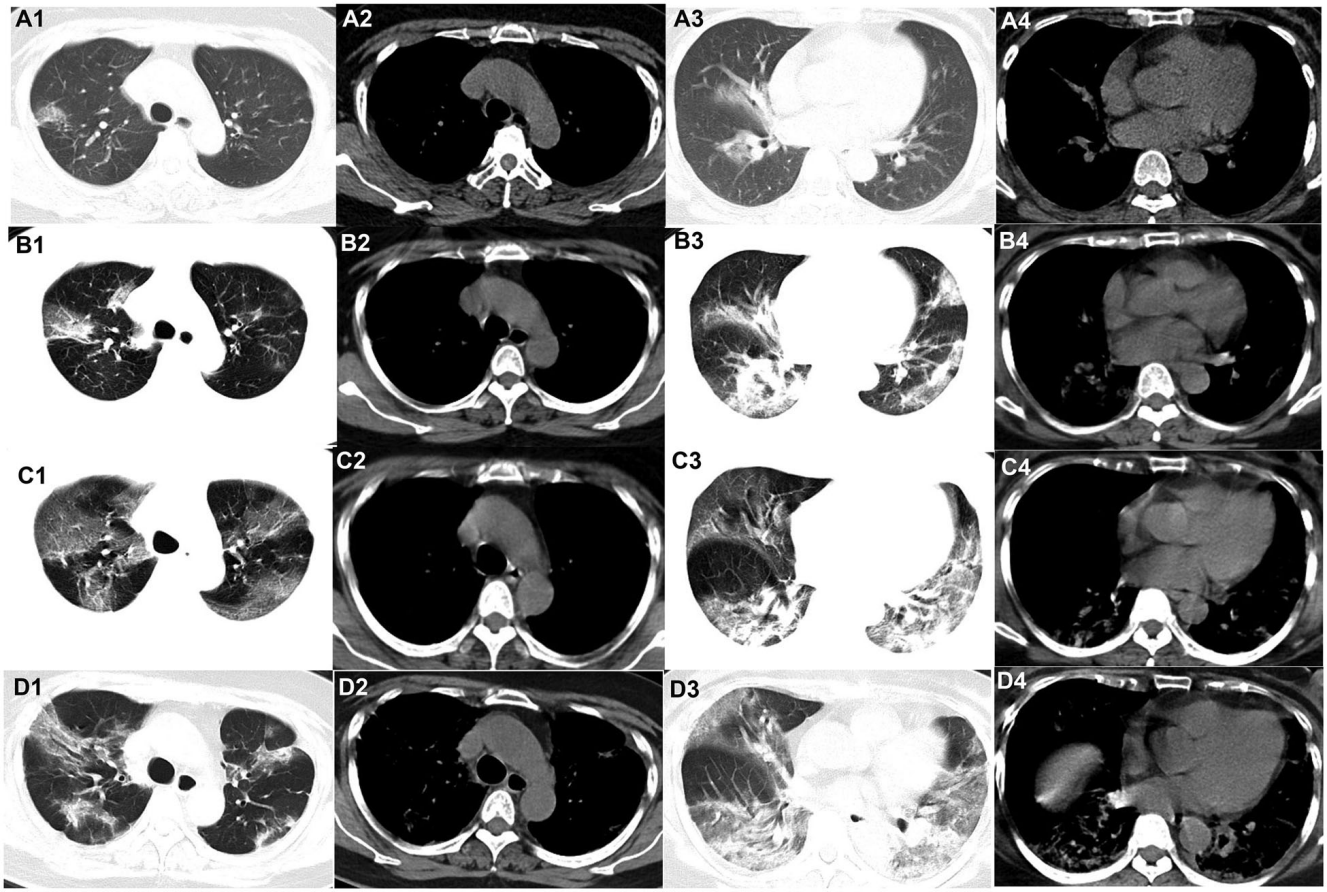
Evolution of the pure ground-glass opacities (GGO) on chest CT of patients with COVID-19. A 58-year-old woman who had close contact with individuals in Wuhan presented with fever for 2 days. (A1–A4) The first non-contrast-enhanced chest CT reveals multipleground-glass opacities in the right upper, middle, and lower lobes (initial chest CT). (B1–B4) Follow-up chest CT 4 days after the first shows that both the scope and density of the lesions increase (stage I*). (C1–C4) Follow-up chest CT 8 days after the first shows that the scope of the lesions increases while the density decreases (stage II*). (D1–D4) Follow-up chest CT 13 days after the first shows that the scope of the lesions decreases slightly while the density increases (stage III*). *The stage does not represent the course of COVID-19 but the time interval between the two adjacent CT scans.

**Figure 2.**
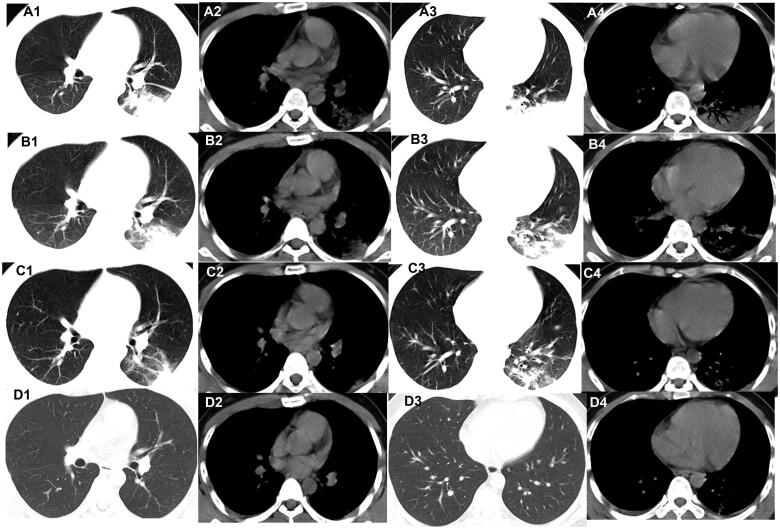
Evolution of consolidationon chest CT of patients with COVID-19. A 45-year-old male Wuhan resident presented with fever and cough for 3 days. (A1–A4) The first non-contrast-enhanced chest CT revealsconsolidation and air bronchogram in the left lower lobe (initial chest CT). (B1–B4) Follow-up chest CT 4 days after the first shows that both the scope and density of the lesions decrease (stage I*). (C1–C4) Follow-up chest CT 8 days after the first shows that both the scope and density of the lesions decrease further (stage II*). (D1–D4) Follow-up chest CT 12 days after the first shows that the lesions are almost absorbed completely (stage III*). *The stage does not represent the course of COVID-19 but the time interval between the two adjacent CT scans.

**Figure 3.**
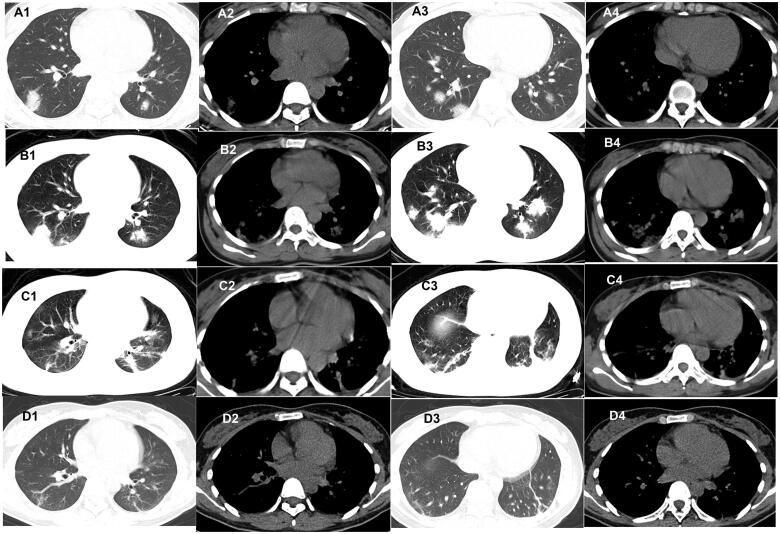
Evolution of the nodules on chest CT of patients with COVID-19. A 27-year-old woman who had close contact with individuals in Wuhan presented with fever and fatigue for 3 days. (A1–A4) The first non-contrast-enhanced chest CT reveals multiple nodules in the left and right lower lobes (initial chest CT). (B1–B4) Follow-up chest CT 4 days after the first shows that both the scope and density of the lesions increase (stage I*); (C1–C4) Follow-up chest CT 8 days after the first shows that both the scope and density of the lesions decrease (stage II*). (D1–D4) Follow-up chest CT 12 days after the first shows that the lesions are almost absorbed completely (stage III*). *The stage does not represent the course of COVID-19 but the time interval between the two adjacent CT scans.

**Figure 4.**
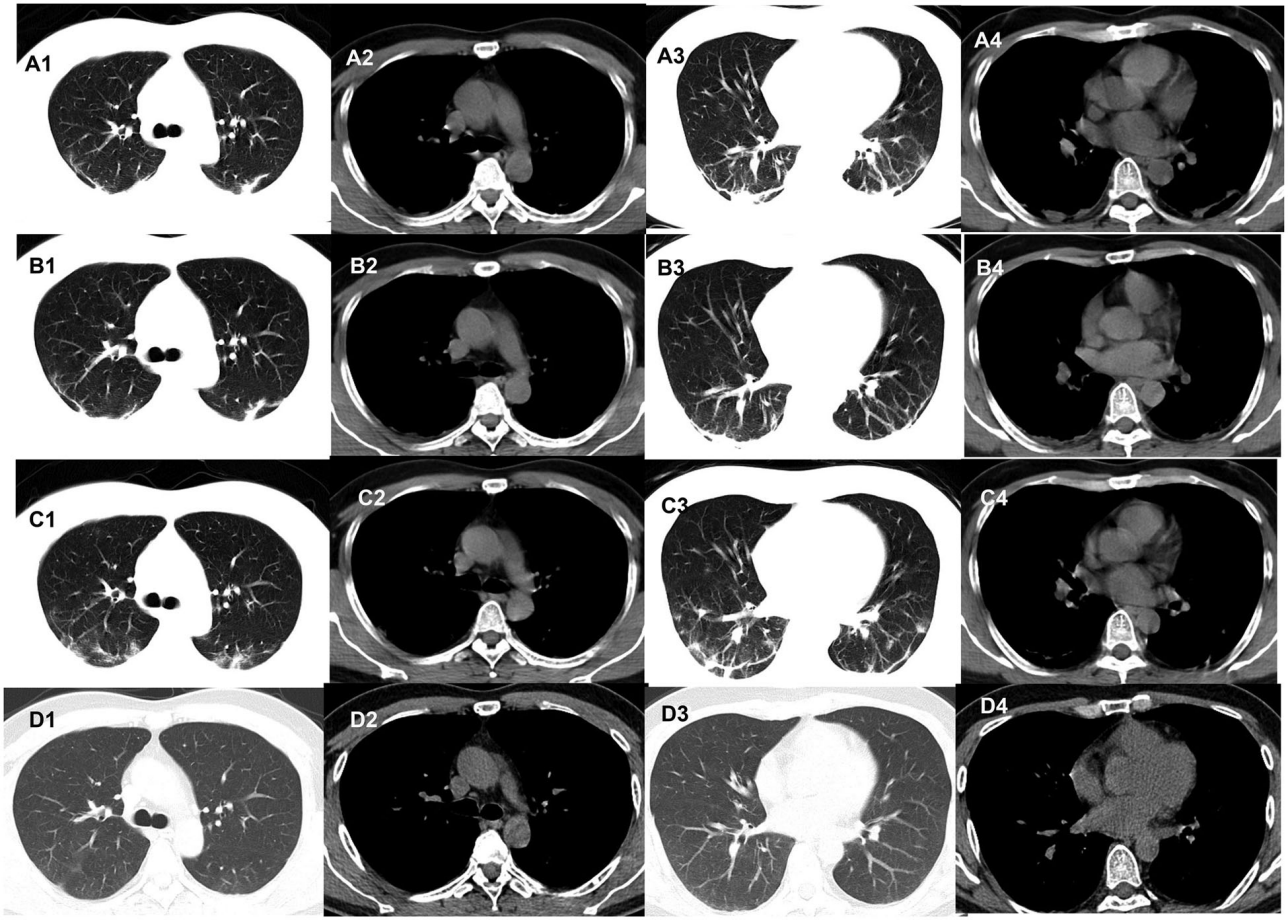
Evolution of the fibrous stripes on chest CT of patients with COVID-19. A 42-year-old man who had close contact with individuals in Wuhan presentedwith fever for 1 day. (A1–A4) The first non-contrast-enhanced chest CT reveals multiple fibrous stripes in the left and right lower lobes (initial chest CT). (B1–B4)Follow-up chest CT 4 days after the first shows that both the scope and density of the lesions decrease (stage I*). (C1–C4) Follow-up chest CT 8 days after the first shows that both the scope and density of the lesions decrease further (stage II*). (D1–D4) Follow-up chest CT 12 days after the first shows that the lesions are almost absorbed completely (stage III*). *The stage does not represent the course of COVID-19 but the time interval between the two adjacent CT scans.

**Figure 5.**
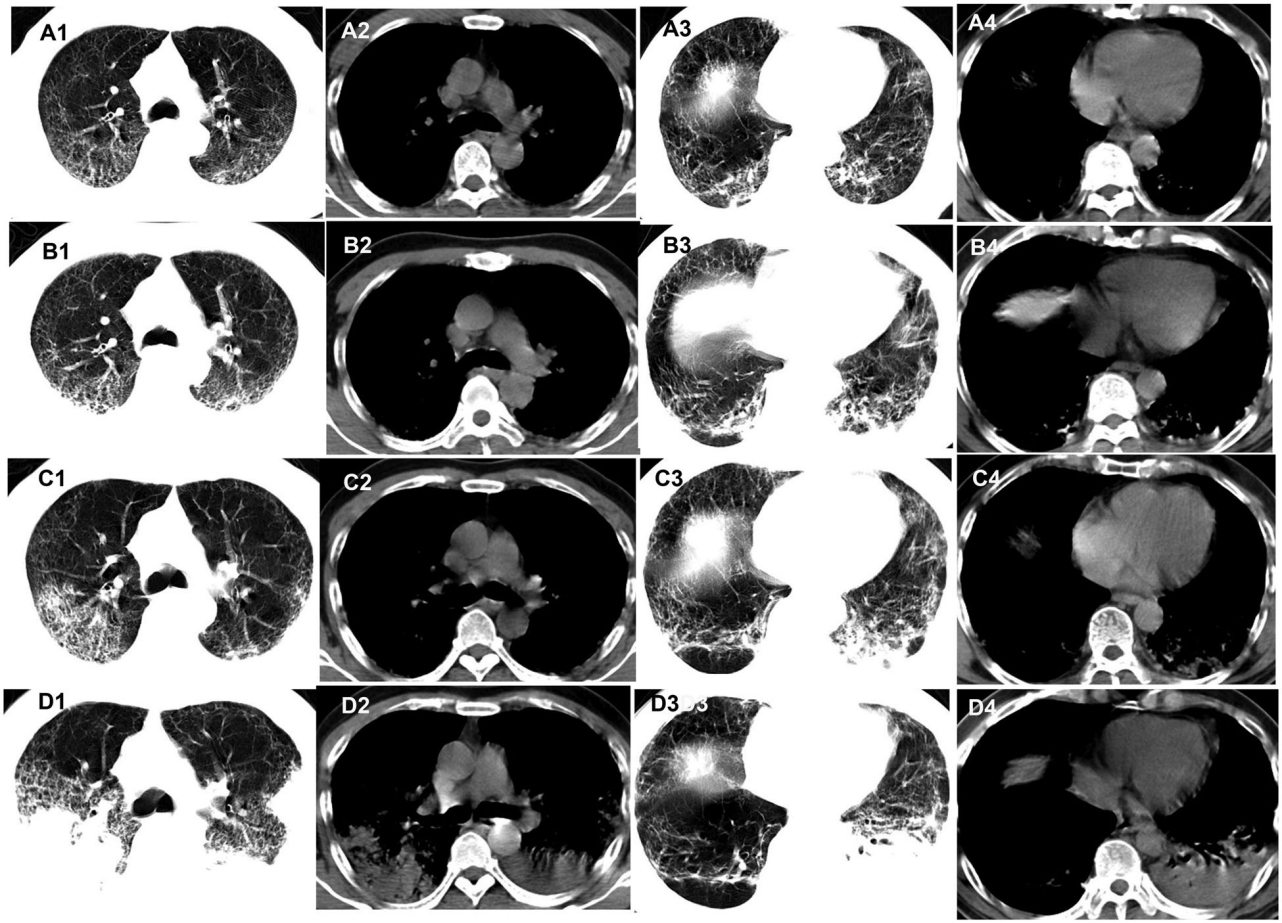
Evolution of the mixed pattern on chest CT of patients with COVID-19. A 64-year-old male Wuhan resident presented with cough, fever, and fatiguefor 3 days. (A1–A4) The first non-contrast-enhanced chest CT scan reveals multiple ground-glass opacities (GGO), fibrous stripes and visible interlobular septal thickening in the left and right upper and lower lobes (initial chest CT). (B1–B4) Follow-up chest CT 4 days after the first shows that both the scope and density of the lesions increase (stage I*). (C1–C4) Follow-up chest CT 8 days after the first shows that both the scope and density of the lesions increase further (stage II*). (D1–D4) Follow-up chest CT 12 days after the first shows that both the scope and density of the lesions increase even further (stage III*). *The stage does not represent the course of COVID-19 but the time interval between the two adjacent CT scans.

By 20 February 2020, 361 patients who were admitted to Wuhan First Hospital (*n* = 309) or Xiangya Hospital (*n* = 52) and who met the inclusion criteria were retrospectively enrolled in our study. Among them, 103 (28.5%) patients had pure GGO patterns of initial CT manifestations, 83 (23.0%) patients had solid nodules, 47 (13.0%) patients had consolidation, and 19 (5.3%) patients had fibrous stripes. However, a large proportion (109, 30.2%) had mixed patterns (two or more than two types of the four morphological lesions [GGO, consolidation, nodule, and fibrous stripes]). Regarding age, 107 (29.6%) patients were aged 30–44 years; 101 (28.0%) were aged ≥60 years, 89 (24.7%) were aged 45–59 years, 43 (11.9%) were aged 14–29 years and 21 (5.8%) were aged < 14 years. Compared with the other CT patterns, nodules were more common (20.5%) in the group aged 14–29 years, and fibrous stripe (47.4%) and nodules (41.0%) were more common in the group aged 30–44years. However, among those aged older than 60 years, mixed (39.4%) and pure GGO (30.1%) were more common than the other patterns (Table 1, Figures 1–5).

**Table 1.** Clinical characteristics of 361 patients with COVID-19 pneumonia at presentation.

Variable	All patients	Pure GGO	Consolidation	Nodules	Fibrous stripes	Mixed	*p* Value
Number of patients, No. (%)	361 (100)	103 (28.5)	47 (13.0)	83 (23.0)	19 (5.3)	109 (30.2)	
Age groups, No. (%)							
<14 years	21 (5.8)	8 (7.8)	2 (4.3)	4 (4.8)	0 (0)	7 (6.4)	.677
14–29 years	43 (11.9)	13 (12.6)	1 (2.1)	17 (20.5)	1 (5.3)	11 (10.1)	.023
30–44 years	107 (29.6)	23 (22.3)	16 (34.0)	34 (41.0)	9 (47.4)	25 (22.9)	.010
45–59 years	89 (24.7)	28 (27.2)	14 (29.8)	19 (22.9)	5 (26.3)	23 (21.1)	.749
≥60 years	101 (28.0)	31 (30.1)	14 (29.8)	9 (10.8)	4 (21.1)	43 (39.4)	.001
Male, No. (%)	193 (53.0)	48 (46.6)	24 (51.1)	51 (61.4)	11 (57.9)	59 (54.1)	.360
Exposure to source of transmission within 14 days, No. (%)							
Come from Wuhan	304 (84.2)	84 (81.6)	39 (83.0)	72 (86.7)	14 (73.7)	95 (87.2)	.509
Recently been to Wuhan	16 (4.4)	5 (4.9)	2 (4.3)	3 (3.6)	2 (10.5)	4 (3.7)	.737
Contacted with people from Wuhan	24 (6.6)	10 (9.7)	3 (6.4))	4 (4.8))	2 (10.5))	5 (4.6)	.523
Contacted with confirmed cases	131 (36.3)	47 (45.6)	14 (29.8)	26 (31.3)	6 (31.6)	38 (34.9)	.206
No exposure history	17 (4.7)	4 (3.9)	3 (6.4)	4 (4.8)	1 (5.3)	5 (4.6)	.976
Familial cluster, No. (%)	65 (18.0)	21 (20.4)	9 (19.1)	13 (15.7)	5 (26.3)	17 (15.6)	.724
Smoking history, No. (%)							
Never smokers	301 (83.4)	85 (82.5)	39 (83.0)	74 (89.2)	16 (84.2)	87 (79.8)	.547
Ex-smokers	19 (5.3)	7 (6.8)	2 (4.3)	1 (1.2)	1 (5.3)	8 (7.3)	.371
Current smokers	41 (11.4)	11 (10.7)	6 (12.8)	8 (9.6)	2 (10.5)	14 (12.8)	.959
Coexisting conditions, No. (%)							
Hypertension	45 (12.5)	13 (12.6)	7 (14.9)	10 (12.0)	2 (10.5)	13 (11.9)	.985
Diabetes	17 (4.7)	4 (3.9)	5 (4.6)	0 (0)	1 (5.3)	7 (6.4)	.070
Chronic obstructive pulmonary diseases	32 (8.6)	11 (10.7)	6 (12.8)	1 (1.2)	1 (5.3)	12 (11.0)	.076
Coronary heart disease	13 (3.6)	4 (3.9)	3 (6.4)	0 (0)	1 (5.3)	5 (4.6)	.328
Cerebrovascular diseases	9 (2.5)	4 (3.9)	2 (4.3)	0 (0)	0 (0)	3 (2.8)	.398
Renal disease	3 (0.8)	1 (1.0)	1 (2.1)	0 (0)	0 (0)	1 (0.9)	.764
Hepatobiliary disease	16 (4.4)	5 (4.9)	3 (6.4)	1 (1.2)	1 (5.3)	6 (5.5)	.586
Cancers	2 (0.6)	0 (0)	0 (0)	0 (0)	1 (5.3)	1 (0.9)	.056
Hematological tumours	2 (0.6)	1 (1.0)	0 (0)	0 (0)	1 (5.3)	0 (0)	.054
Graves’ disease	2 (0.6)	1 (1.0)	0 (0)	0 (0)	0 (0)	1 (0.9)	.841
Rheumatic connective tissue disease	3 (0.8)	1 (1.0)	1 (2.1)	0 (0)	0 (0)	1 (0.9)	.764
Congenital heart disease	4 (1.1)	1 (1.0)	1 (2.1)	0 (0)	1 (5.3)	1 (0.9)	.352
Signs and symptoms, No. (%)							
Fever on admission							
Temperature on admission, °C							
<37.3	197 (54.6)	57 (55.3)	24 (55.1)	61 (73.5)	12 (63.2)	43 (39.4)	.000
37.3–38.0	108 (29.9)	29 (28.2)	11 (23.4)	19 (22.9)	5 (26.3)	44 (40.4)	.065
38.1–39.0	37 (10.2)	13 (12.6)	7 (14.9)	2 (2.4)	1 (5.3)	14 (12.8)	.072
>39.0	19 (5.3)	4 (3.9)	5 (10.6)	1 (1.2)	1 (5.3)	8 (7.3)	.147
Fever during hospital admission							
Highest temperature during hospital admission, °C							
<37.3	74 (20.5)	19 (18.4)	4 (8.5)	41 (49.4)	6 (31.6)	4 (3.7)	.000
37.3–38.0	101 (28.0)	32 (31.1)	11 (23.4)	35 (42.2)	9 (47.4)	14 (12.8)	.000
38.1–39.0	117 (32.4)	34 (29.1)	19 (16.2)	5 (6.0)	2 (10.5)	57 (52.3)	.000
>39.0	69 (19.1)	18 (17.5)	13 (27.7)	2 (2.4)	2 (10.5)	34 (31.2)	.000
Cough	237 (65.7)	65 (63.1)	34 (72.3)	54 (65.1)	13 (68.4)	71 (65.1)	.858
Sputum production	121 (33.5)	12 (11.7)	31 (66.0)	9 (10.8)	2 (10.5)	67 (61.5)	.000
Dyspnoea	65 (18.0)	25 (24.3)	11 (23.4)	1 (1.2)	1 (5.3)	27 (24.8)	.000
Hemoptysis	2 (0.6)	0 (0)	1 (2.1)	0 (0)	0 (0)	1 (0.9)	.475
Sore throat	46 (12.7)	21 (20.4)	3 (6.4)	9 (10.8)	1 (5.3)	12 (11.0)	.071
Diarrhea	13 (3.6)	3 (2.9)	2 (4.3)	3 (3.6)	1 (5.3)	4 (3.7)	.986
Nausea or vomiting	7 (1.9)	1 (1.0)	2 (4.3)	1 (1.2)	0 (0)	3 (2.8)	.588
Fatigue	74 (20.5)	23 (22.3)	15 (31.9)	7 (8.4)	3 (15.8)	26 (23.9)	.015
Myalgia or arthralgia	38 (10.5)	11 (10.7)	9 (19.1)	3 (3.6)	1 (5.3)	14 (12.8)	.059
Headache	11 (3.0)	4 (3.9)	3 (6.4)	0 (0)	0 (0)	4 (3.7)	.252
Chill	56 (15.5)	13 (12.6)	12 (25.5)	1 (1.2)	1 (5.3)	29 (26.6)	.000
Respiratory rate >24 breaths per min	49 (13.6)	17 (16.5)	8 (17.0)	0 (0)	1 (5.3)	23 (21.1)	.000

GGO: ground-glass opacities.

Of the 361 patients, 193 (53.0%) were men, 304 (84.2%) resided in Wuhan, 16 (4.4%) had recently travelled to Wuhan before disease onset, and 24 (6.6%) had contact with individuals from Wuhan. Moreover, 131 (36.3%) had contact with confirmed cases, and 65 (18.0%) were family cluster cases. However, no significant differences were found among the different chest CT patterns with respect to sex, epidemiological history, or underlying comorbidities (*p* > .05) (Table 1). Fever and cough were the most common symptoms. Fever, dyspnoea, and a respiratory rate >24 breaths per min were significantly more common in the group with mixed and pure GGO patterns (*p* < .01). Sputum production and chills were significantly more common in the group with mixed and consolidation patterns (*p* < .01) (Table 1).

### Laboratory, image findings and outcomes

Regarding laboratory findings, leucopoenia (white blood cell count <4 × 10^9^/L) and lymphopenia (<1.0 × 10^9^/L) were more commonly observed in patients with pure GGO (62.1, 47.6%, respectively). Leukocytosis (white blood cell count >10 × 10^9^/L) was more common in patients with consolidation (29.8%). Additionally, increased levels of alanine aminotransferase (ALT) (>40 U/L), aspartate aminotransferase (AST) (>40 U/L), total bilirubin (>17.1 mmol/L), creatine kinase (>185 U/L), C-reactive protein (CRP) (>10 mg/L), and the erythrocyte sedimentation rate (ESR) (>20 mm/h) were more common in patients with mixed patterns. Increased procalcitonin levels (≥0.5 ng/mL) were more common in patients with consolidation. A decreased oxygen index was more common in patients with pure GGO, mixed and consolidation patterns, while a normal oxygen index was more common in patients with nodules and fibrous stripes (Table 2).

**Table 2.** Laboratory findings, image findings, complications, treatment, and outcomes in 361 patients with COVID-19 pneumonia.

Variable	All patients	Pure GGO	Consolidation	Nodules	Fibrous stripes	Mixed	*p* Value
Laboratory findings							
White blood cell count (×10^9^/L)	5.1 (1.3–23.9)	4.6(1.3–17.4)	8.4(3.2–22.5)	4.8(3.1–12.6)	6.1(3.5–9.9)	4.9(2.6–23.9)	
<4, No. (%)	146 (40.4)	64 (62.1)	4 (8.5)	29 (34.9)	8 (42.1)	41 (37.6)	.000
4–10, No. (%)	172 (47.6)	34 (33.0)	29 (61.7)	53 (63.9)	11 (57.9)	45 (41.3)	.000
>10, No. (%)	43 (11.9)	5 (4.9)	14 (29.8)	1 (1.2)	0 (0)	23 (21.1)	.000
Neutrophil count (×10^9^ / L)	4.1 (0.7–18.7)	3.3 (0.7–14.1)	4.8 (1.9–18.7)	3.7 (1.1–12.8)	3.9 (0.8–11.9)	4.5 (0.9–17.7)	
Lymphocyte count (×10^9^ / L)	0.9 (0.3–3.1)	0.8 (0.3–1.9)	1.1 (0.7–2.8)	1.2 (0.4–2.3)	0.9 (0.5–3.1)	0.8 (0.4–2.7)	
<10, No. (%)	124 (34.3)	49 (47.6)	7 (14.9)	23 (27.7)	4 (21.1)	41 (37.6)	.001
Platelet count (×10^9^ / L)	174.8 (21.0–463.0)	168.7 (21.0–426)	187.3 (79.0–463.0)	177.5 (29.0–358.0)	171.3 (31.0–327.0)	176.4 (46.0–439.0)	
<100, No. (%)	27 (7.5)	9 (8.7)	6 (12.8)	1 (1.2)	0 (0)	11 (10.1)	.050
Hemoglobin, g/L	131.4 (68.0–173)	127.6 (76.0–165.0)	124.1 (68.0–152.0)	133.5 (83.0–154.0)	132.7 (79.0–161.0)	129.4 (69.0–173.0)	
Alanine aminotransferase (U/L)	31.8 (8.3–134.9)	34.5 (13.3–126.7)	33.7 (15.4–118.7)	23.1 (8.3–84.1)	25.2 (11.5–66.1)	35.2 (26.3–134.9)	
>40, No. (%)	31 (8.6)	9 (8.7)	3 (6.4)	1 (1.2)	1 (5.3)	17 (15.6)	.011
Aspartate aminotransferase (U/L)	29.8 (11.1–121.3)	31.4 (11.1–116.8)	30.5 (14.2–121.3)	22.7 (13.8–93.4)	26.1 (18.2–70.6)	32.8 (21.2–118.6)	
>40, No. (%)	42 (11.6)	11 (10.7)	5 (10.6)	2 (2.4)	1 (5.3)	23 (21.1)	.002
Total bilirubin (mmol / L)	12.3 (4.7–51.1)	14.1 (5.2–43.6)	17.4 (4.9–48.5)	11.3 (4.7–32.6)	11.4 (4.8–30.5)	12.2 (6.9–51.1)	
>17.1, No. (%)	39 (10.8)	12 (11.7)	4 (8.5)	1 (1.2)	1 (5.3)	21 (19.3)	.002
Creatinine (μmol / L)	87.4 (21.3–374.8)	104.6 (28.5–346.8)	113.1 (34.7–362.5)	51.3 (21.3–171.9)	62.4 (31.8–247.3)	98.1 (26.2–374.8)	
>133, No. (%)	14 (3.9)	5 (4.9)	1 (2.1)	1 (1.2)	0 (0)	7 (6.4)	.298
Creatine kinase (U / L)	137.6	134.9	162.5	93.4	116.7	148.5	
	(10.1–974.6)	(23.1–651.8)	(47.1–836.2)	(10.1–295.7)	(38.2–371.9)	(50.4–974.6)	
>185, No. (%)	51 (14.1)	13 (12.6)	4 (8.5)	6 (7.2)	2 (10.5)	26 (23.9)	.010
Lactate dehydrogenase (U/L)	231.5	246.3	253.6	179.3	184.4	252.4	
	(101.4–573.8)	(108.3–514.2)	(130.5–569.1)	(101.4–397.2)	(124.7–425.6)	(143.6–573.8)	
>250, No. (%)	83 (23.0)	24 (23.3)	7 (14.9)	16 (19.3)	5 (36.3)	31 (28.4)	.362
D-dimer (mg/L)	0.6(0.01–9.18)	0.5(0.02–7.78)	0.6(0.04–8.05)	0.3(0.01–5.41)	0.4(0.03–4.37)	0.7(0.02–9.18)	
≥0.5, No. (%)	74 (20.5)	19 (18.4)	8 (17.0)	19 (22.9)	4 (21.1)	24 (22.0)	.900
C-reactive protein level (mg/L)	(1.71–119.8)	(1.87–101.9)	(8.65–108.4)	(1.71–69.2)	(4.25–76.3)	(7.81–119.8)	
>10, No. (%)	253 (70.1)	64 (62.1)	31 (66.0)	59 (71.1)	8 (42.1)	91 (83.5)	.001
Erythrocyte sedimentation rate (mm / h)	57.6(5.0–147.0)	58.3(11.0–134.0)	61.6(13.0–144.0)	49.4(5.0–117.0)	53.2(7.0–121.0)	59.8(9.0–147.0)	
>20, No. (%)	274 (75.9)	69 (67.0)	35 (74.5)	62 (74.7)	11 (57.9)	97 (89.0)	.001
Procalcitonin (ng / mL)	0.14(0.00–4.70)	0.09(0.00–3.21)	0.16(0.00–4.70)	0.06(0.00–0.49)	0.05(0.00–0.47)	0.15(0.00–4.51)	
≥0.5, No. (%)	21 (5.8)	3 (2.9)	7 (14.9)	0 (0)	0 (0)	11 (10.1)	.001
Oxygen index							
>300	191 (52.9)	45 (43.7)	16 (34.0)	81 (97.6)	15 (78.9)	34 (31.2)	.000
200–300	74 (20.5)	25 (24.3)	13 (27.7)	2 (2.4)	3 (15.8)	31 (28.4)	.000
100–200	49 (13.6)	19 (18.4)	11 (23.4)	0 (0)	1 (5.3)	18 (16.5)	.000
<100	47 (13.0)	14 (13.6)	7 (14.9)	0 (0)	0 (0)	26 (23.9)	.000
Imaging findings, No. (%)							
Distribution							
** **Unilateral	88 (24.4)	22 (21.4)	15 (31.9)	13 (15.7)	17 (89.5)	21 (19.3)	.000
** **Bilateral	273 (75.6)	81 (78.6)	32 (68.1)	70 (84.3)	2 (10.5)	88 (80.7)	.000
Central	3 (0.8)	2 (1.9)	0 (0)	1 (0)	0 (0)	0 (0)	.533
Peripheral	303 (83.9)	87 (84.5)	31 (66.0)	77 (92.8)	18 (94.7)	90 (82.6)	.001
Both central and peripheral	55 (15.2)	14 (13.6)	16 (34.0)	5 (6.0)	1 (5.3)	19 (17.4)	.000
Left upper lobe	108 (29.9)	37 (35.9)	8 (17.0)	31 (37.3)	4 (21.1)	28 (25.7)	.053
Left lower lobe	189 (52.4)	53 (51.5)	29 (61.7)	43 (51.8)	7 (36.8)	57 (52.3)	.474
Right upper lobe	129 (35.7)	38 (36.9)	11 (23.4)	34 (41.0)	5 (26.3)	41 (37.6)	.281
Right middle lobe	52 (14.4)	14 (13.6)	6 (12.8)	12 (14.5)	2 (10.5)	18 (16.5)	.941
Right lower lobe	231 (64.0)	64 (62.1)	35 (74.5)	51 (61.4)	8 (42.1)	73 (67.0)	.136
Extent of lesion involvement							
Focal	49 (13.6)	13 (12.6)	8 (17.0)	9 (10.8)	15 (78.9)	4 (3.7)	.000
Multifocal	249 (69.0)	69 (67.0)	30 (63.8)	74 (89.2)	4 (21.1)	72 (66.1)	.000
Diffuse	63 (17.5)	21 (20.4)	9 (19.1)	0 (0)	0 (0)	33 (17.5)	.000
Complications, No. (%)							
ARDS	49 (13.6)	14 (13.6)	8 (17.0)	0 (0)	0 (0)	27 (24.8)	.000
Septic shock	7 (1.9)	2 (1.9)	2 (4.3)	0 (0)	0 (0)	3 (2.8)	.445
AKI	6 (1.7)	2 (1.9)	2 (4.3)	0 (0)	0 (0)	2 (1.8)	.444
DIC	4 (1.1)	2 (1.9)	0 (0)	0 (0)	0 (0)	2 (1.8)	.584
Rhabdomyolysis	51 (14.1)	13 (12.6)	4 (8.5)	6 (7.2)	2 (10.5)	26 (23.9)	.010
Treatments, No. (%)							
Antiviral therapy	361 (100)	103 (100)	47 (100)	83 (100)	19 (100)	109 (100)	
Antimicrobial therapy	119 (33.0)	34 (33.0)	17 (36.2)	7 (8.4)	3 (15.8)	58 (53.2)	.000
Use of corticosteroid	29 (8.0)	11 (10.7)	3 (6.4)	0 (0)	0 (0)	15 (13.8)	.005
CRRT	38 (10.5)	13 (12.6)	4 (8.5)	0 (0)	0 (0)	21 (19.3)	.000
Oxygen therapy							
Nasal cannula	137 (38.0)	31 (30.1)	15 (31.9)	34 (41.0)	11 (57.9)	46 (42.2)	.100
High-flow nasal cannula	65 (18.0)	25 (24.3)	13 (27.7)	0 (0)	0 (0)	27 (24.8)	.000
Non-invasive ventilation	26 (7.2)	9 (8.7)	4 (8.5)	0 (0)	0 (0)	13 (11.9)	.017
Invasive mechanical ventilation	21 (5.8)	7 (6.8)	3 (6.4)	0 (0)	0 (0)	11 (10.1)	.038
Severe, No. (%)	46 (12.7)	11 (10.7)	8 (17.0)	2 (2.4)	2 (10.5)	23 (21.1)	.003
Prognosis, No. (%)							
Discharge	332 (92.0)	94 (91.3)	44 (93.6)	83 (100.0)	19 (100.0)	92 (84.4)	0.001
Death	29 (8.0)	9 (8.7)	3 (6.4)	0 (0)	0 (0)	17 (15.6)	0.001

GGO: ground-glass opacities; ARDS: acute respiratory distress syndrome; AKI: acute kidney injury; DIC: disseminated intravascular coagulation; CRRT: continuous renal replacement therapy.

Chest CT scans revealed that most of the lesions were distributed bilaterally (75.6%) and peripherally (83.9%) (Table 2). Fibrous stripes were more commonly distributed unilaterally and peripherally. All the lesions were more commonly distributed in the right lower lobe (64%). Additionally, fibrous stripes were more commonly focal (78.9%), while nodules were multifocal (89.2%).

Forty-six patients had severe cases of the disease (46/361; 12.7%). Severe cases were most often associated with mixed CT manifestations (21.1%), followed by the consolidation pattern (17.0%) and less often with nodules (2.4%). The most common complications were rhabdomyolysis (14.1%), followed by acute respiratory distress syndrome (ARDS) (13.6%). All the patients received antiviral therapy, and 119 (33.0%) were managed with empirical antibiotic therapy. Antimicrobial therapy was more commonly administered to patients with mixed (53.2%) and consolidation (36.2%) patterns. Moreover, corticosteroids were administered to 29 (8.0%) patients, and 38 (10.5%) received continuous renal replacement therapy; both treatments were prescribed more commonly to patients with mixed (13.8 and 19.3%, respectively) and pure GGO (10.7 and 12.6%, respectively) patterns. Additionally, high-flow nasal cannula therapy, non-invasive ventilation, and invasive mechanical ventilation were initiated in 18.0, 7.2, and 5.8% of all patients, respectively (Table 2). None of these treatments were initiated in patients with nodules and fibrous stripes. In total, 332 (92.0%) patients were discharged, and 29 (8.0%) died. None of the deceased patients were in the group with nodules and fibrous stripes (Table 2).

### Follow-up chest CT outcomes

For stage I disease (between the first and second chest CT), the chest CT images of 43 patients (11.9%) showed no significant changes. Disease progression was observed in 161 cases (44.6%), and 81 patients (22.4%) presented with absorbed lesions. The lesions of 76 patients (21.1%) disappeared in one area but appeared at another site. None of the patients presented with pleural effusion or fibrosis in stage I. Additionally, patients with nodules were found to experience progression less frequently than those with a mixed pattern or pure GGO or consolidation. Regarding the manner of progression, only enlargement of the lesions with no change in density was more common in patients with fibrous stripes or pure GGO than in patients with the other patterns. Both increases in the density and size were more common in patients with the consolidation or mixed pattern. The appearance of new lesions was more common in patients with nodules and fibrous stripes (*p* < .01). The disappearance of lesions in one area but their appearance at another site was more common in patients with nodules than in those with a mixed pattern or pure GGO. Regarding absorption, reduction in density but slight increase in size was more common in patients with nodules (*p* < .01) (Table 3). For stage II disease (between the second and third chest CT), stable lesions and progression of the lesions were observed in 31 (8.6%) and 95 (44.6%) cases, respectively, less than those observed in stage I. However, 176 patients (48.8%) presented with absorbed lesions, more than those with stage I disease. The lesions of 59 patients (16.3%) disappeared in one area and appeared at another site. Two patients had pleural effusion, and 1 patient showed fibrosis. In stage III (between the third and fourth chest CT), stable lesions and progression of the lesions were observed in 13 (3.6%) and 29 (8.0%) cases, respectively, less than those observed in those stage I/II disease. Most of the patients (305, 84.5%) presented with absorbed lesions, more than those in stages I/II disease. The lesions of 2 patients (16.3%) disappeared in one area but appeared at another site. Two patients showed pleural effusion, and 3 patients had fibrosis. The manner of progression and absorption of different chest CT patterns among those with stage II and III disease was similar to that observed among those with stage I disease. The medium TSS in the group with nodules and fibrous stripes was significantly lower than that in the group with mixed patterns in all three stages (*p* < .01) (Table 3).

**Table 3.** The follow-up results of chest CT in 361 patients with COVID-19 pneumonia.

Variable		All patients	Pure GGO	Consolidation	Nodules	Fibrous stripes	Mixed	*p* Value
Stage I		361	103	47	83	19	109	
Stable		43 (11.9)	11 (10.7)	8 (17.0)	14 (16.9)	3 (15.8)	7 (6.4)	.155
Progression		161 (44.6)	53 (51.5)	24 (51.1)	12 (14.5)	8 (42.1)	64 (58.7)	.000
Density	Scope							
↑	–	22 (13.7)	4 (7.5)	3 (12.5)	1 (8.3)	0 (0)	14 (21.9)	.140
–	↑	32 (19.9)	19 (35.8)	2 (8.3)	1 (8.3)	3 (37.5)	7 (10.9)	.003
↑	↑	45 (28.0)	7 (13.2)	11 (45.8)	1 (8.3)	0 (0)	26 (40.6)	.000
Appearance of new lesions		62 (38.5)	23 (43.4)	8 (33.3)	9 (75.0)	5 (62.5)	17 (26.6)	.008
Disappearance in one area but appearance at another site		76 (21.1)	17 (16.5)	3 (6.4)	31 (37.3)	2 (10.5)	23 (21.1)	.000
Absorption		81 (22.4)	22 (21.4)	12 (25.5)	26 (31.3)	6 (31.6)	15 (13.8)	.045
Density	Scope							
↓	–	21 (25.9)	5 (22.7)	4 (33.3)	4 (15.4)	1 (16.7)	7 (46.7)	.232
–	↓	21 (25.9)	14 (63.6)	2 (16.7)	0 (0)	3 (50.0)	2 (13.3)	.000
↓	↓	23 (28.4)	3 (13.6)	6 (50.0)	7 (26.9)	2 (33.3)	5 (33.3)	.249
↓	slight ↑	16 (19.8)	0 (0)	0 (0)	15 (93.8)	0 (0)	1 (6.7)	.000
Appearance of pleural effusion		0 (0)	0 (0)	0 (0)	0 (0)	0 (0)	0 (0)	
Appearance of fibrosis		0 (0)	0 (0)	0 (0)	0 (0)	0 (0)	0 (0)	
Medium TSS		4 (1–8)	4 (1–6)	5 (2–6)	3 (1–4)	3 (1–5)	5 (3–8)	.000
Stage II								
Stable		31 (8.6)	7 (6.8)	5 (10.6)	11 (13.3)	2 (10.5)	6 (5.5)	.356
Progression		95 (26.3)	27 (26.2)	13 (27.7)	9 (10.8)	5 (26.3)	41 (37.6)	.002
Density	Scope							
↑	–	13 (13.7)	2 (7.4)	2 (15.4)	0 (0)	0 (0)	9 (22.0)	.238
–	↑	22 (23.2)	13 (48.1)	1 (7.7)	1 (11.1)	3 (60.0)	4 (9.8)	.001
↑	↑	29 (30.5)	1 (3.7)	7 (53.8)	1 (11.1)	0 (0)	20 (48.8)	.000
Appearance of new lesions		31 (32.6)	11 (40.7)	3 (23.1)	7 (77.8)	2 (40.0)	8 (19.5)	.011
Disappearance in one area but appearance at another site		59 (16.3)	13 (12.6)	2 (4.3)	27 (32.5)	1 (5.3)	16 (14.7)	.000
Absorption		176 (48.8)	56 (54.4)	27 (57.4)	36 (43.4)	11 (57.9)	46 (42.2)	.185
Density	Scope							
↓	–	50 (28.4)	11 (19.6)	9 (33.3)	11 (30.6)	1 (9.1)	18 (39.1)	.129
–	↓	41 (23.3)	29 (51.8)	4 (14.8)	0 (0)	5 (45.5)	3 (6.5)	.000
↓	↓	71 (40.3)	16 (28.6)	14 (51.9)	13 (36.1)	5 (45.5)	23 (50.0)	.142
↓	slight ↑	14 (8.0)	0 (0)	0 (0)	12 (85.7)	0 (0)	2 (4.3)	.000
Appearance of pleural effusion		2 (0.6)	0 (0)	1 (2.1)	0 (0)	0 (0)	1 (0.9)	.475
Appearance of fibrosis		1 (0.3)	0 (0)	0 (0)	0 (0)	1 (5.3)	0 (0)	.001
Medium TSS		8 (4–18)	8 (6–14)	8 (5–14)	6 (4–11)	7 (5–13)	10 (8–18)	.000
Stage III								
Stable		13 (3.6)	3 (2.9)	2 (4.3)	4 (4.8)	1 (5.3)	3 (2.8)	.920
Progression		29 (8.0)	9 (8.7)	3 (6.4)	0 (0)	0 (0)	17 (15.6)	.001
Density	Scope							
↑	–	5 (17.2)	1 (11.1)	1 (33.3)	0 (0)	0 (0)	3 (17.6)	.676
–	↑	6 (20.7)	5 (55.6)	0 (0)	0 (0)	0 (0)	1 (5.9)	.008
↑	↑	11 (37.9)	1 (11.1)	1 (33.3)	0 (0)	0 (0)	9 (52.9)	.111
Appearance of new lesions		7 (24.1)	2 (22.2)	1 (33.3)	0 (0)	0 (0)	4 (23.5)	.923
Disappearance in one area but appearance at another site		14 (3.9)	4 (3.9)	0 (0)	7 (8.4)	0 (0)	3 (2.8)	.105
Absorption		305 (84.5)	87 (84.5)	42 (89.4)	72 (86.7)	18 (94.7)	86 (78.9)	.258
Density	Scope							
↓	–	91 (29.8)	15 (17.2)	23 (54.8)	27 (37.5)	5 (27.8)	21 (24.4)	.000
–	↓	74 (24.3)	39 (44.8)	3 (7.1)	14 (19.4)	6 (33.3)	12 (14.0)	.000
↓	↓	140 (45.9)	33 (37.9)	16 (38.1)	31 (43.1)	7 (38.9)	53 (61.6)	.015
↓	Slight ↑	0	0	0	0	0	0	
Appearance of pleural effusion		2 (0.6)	0 (0)	1 (2.1)	0 (0)	0 (0)	1 (0.9)	.475
Appearance of fibrosis		3 (0.8)	1 (1.0)	1 (2.1)	0 (0)	0 (0)	1 (0.9)	.764
Medium TSS		3 (4–18)	3 (6–14)	3 (5–14)	2 (0–3)	2 (5–13)	4 (8–18)	.000

GGO: ground-glass opacities; TSS: total severity score. ↑: increase; ↓: decrease.

## Discussion

SARS-CoV-2, a single-stranded RNA virus, invades human cells *via* angiotensin-converting enzyme II (ACE2). Subsequently, it damages the pulmonary interstitium and parenchyma. Recently, various imaging chest CT features were reported to reflect different time courses and disease severities in patients with SARS-CoV-2 infection [8,9]. These findings suggest that, a comprehensive understanding of the evolution of chest CT imaging would be of great help to effectively diagnosis and treat COVID-19. In this study, we assessed the CT findings of 361 confirmed COVID-19 patients in various stages and clarified their correlations with the clinical characteristics and laboratory findings.

In our cohort, the typical patterns of initial CT manifestations in COVID-19 patients were GGO, nodules, consolidation, and fibrous stripes. GGO was the most common imaging finding in our study. Some were pure GGO, while others were accompanied by patterns such as consolidation, reticulation, and/or interlobular septal thickening. These results are consistent with those results of previous reports [10–12]. The underlying pathology of GGO may be attributed to diffuse alveolar wall injury, pulmonary edoema and hyaline membrane formation [13,14]. Nodules were frequently related to viral pneumonia [15]. The occurrence rate of simple or multiple nodules in COVID-19 patients in our study was 23%, which was slightly higher than that reported in previous studies (3–13%). The possible explanation for this discrepancy may be related to different regional and ethnic types. Other diseases, including cryptococcosis, lung cancer and tuberculosis, can also present with nodules. However, their morphological characteristics and progression processes are different because of different pathological mechanisms. The pathological mechanisms of the formation of nodules of COVID-19 remain unknown. They are likely not granulomas, which are characterised by localised infiltration and hyperplasia of immunologically modified macrophages and their derived cells (e.g. epithelioid cells and multinucleated giant cells). They may be formed by the filling of alveoli with various inflammatory cells. Consolidation was also observed in COVID-19 patients, ranked after GGO and nodules in our study. The pathological mechanisms may be associated with alveolar wall collapse and replacement of alveoli with cellular fibromyxoid exudates [13]. Fibrous stripes were also among the common chest CT manifestations of COVID-19 in our patients. This finding was consistent with those of the study by Pan et al. [8], who reported 17% COVID-19 patients with fibrous stripes in their study. However, the formation of fibrous stripes requires further study. Additionally, some uncommon chest initial CT manifestations of COVID-19, such as pulmonary fibrosis, cavitation, enlargement of mediastinal and hilar lymph nodes, pleural effusion, and pleural thickening were reported in some publications [10,16]. Although none were present in the early stages of COVID-19 in our cohort, some could be seen in the later phase, consistent with the findings of a recent report [17].

Regarding the distribution of the lesions, bilateral involvement was common (75.6%). Most of the lesions involved two or more lobes simultaneously (96.4%), while single-lobe involvement was rare (3.6%). The right lower lobe was most commonly involved (64.0%), and the left lower lobe was the least commonly affected (52.4%). Nearly 83.9% of the lesions were distributed in the lung periphery, particularly in the subpleural area. These results were consistent with the findings of previous studies [9,10,16].

The initial CT features of COVID-19 differ across age groups [18,19]. We found that nodules were more common in patients aged 14–44 years. However, in patients older than 60 years, mixed patterns were more common. Those findings were slightly different from those of a report by Song et al., who showed that, in patients younger than 50 years, GGO and consolidation were present in 77 and 23%, respectively. However, in patients older than 50 years, the proportions were 55 and 45%, respectively [12].

Regarding the correlation between initial CT manifestations and disease severity, we found that patients with nodules had mild symptoms and rare dyspnoea. However, those with mixed patterns and consolidation were more common in patients with sputum, leukocytosis, and high levels of procalcitonin, indicating that those patients may also have bacterial infections. Additionally, we observed that mixed patterns were more commonly observed in severe cases, while nodules were less common in severe cases. Therefore, the outcomes of patients with nodules were better than those with a mixed pattern. These conclusions were similar to those of a previous report showing that the most common CT findings of COVID-19 were bilateral and multilobar involvement in severe cases [20]. Nevertheless, the mechanisms of the formation of the CT features in severe cases remains to be further explored. These mechanisms may be related to the degree of infection and autoimmunity.

Various stages of COVID-19 have different chest CT features. In the early stage (fewer than 10 days of symptomatic presentation), most of the lesions progressed. This is consistent with previous reports [8,9]. The disappearance of lesions in one area but their appearance at another site was common in patients with nodules. This phenomenon may be associated with strong immunity, which localised the lesions and led to the absorption of some of the lesions. Additionally, the most common manner of absorption of nodules in COVID-19 patients was the reduction in density but slight increase in size. Moreover, the nodules changed very quickly. They were absorbed completely within 4 to 5 days. This phenomenon was different from the absorption pattern of granulomas, which were absorbed slowly. This discrepancy might be explained by the formation of COVID-19 nodules, which were speculated to be caused by them being filled with inflammatory cells but not exudate. Pure GGO and mixed lesions progressed quickly. This finding was similar to that of a previous study, where GGO decreased with more advanced stages of COVID-19. However, the number of lesions showing consolidation or GGO mixed consolidation increased [21]. Moreover, fibrosis was rarely observed in the present study, even during follow-up CT. This finding was consistent with that of a previous study, showing that patients with fibrosis in follow-up CT were older and had a higher rate of ICU admission than those without fibrosis [21].

Our study had several limitations. First, it was limited by the observational nature of the investigation becauseCOVID-19 is a novel disease. Therefore, there may be deviation in the comparison of the results of CT findings. Second, although we have collected as many as four follow-up CT scans to observe the evolution of COVID-19, long-term radiological follow-up is needed to confirm our findings. Finally, no lung biopsy specimens were available. The correlation between chest CT and histopathologic manifestations needs to be investigated in the future.

## Conclusions

Chest CT manifestations of COVID-19 include an imaging pattern of pure GGO, consolidation, nodules, fibrous stripes, and mixed patterns, with the distribution slightly predominant in the lower lobe and peripheral areas of the lung. Nodules are more common in nonsevere cases and young patients. Nodules can be absorbed in a short period, suggesting that patients with nodules have a favourable prognosis. The mixed pattern is more common in severe cases and usually has a relatively poor outcome.

## Data Availability

The datasets analysed during the current study are available from the corresponding author on reasonable request.

## Abbreviations

ACE2: angiotensin converting enzyme II
ARDS: acute respiratory distress syndrome
ALT: alanine aminotransferase
AST: aspartate aminotransferase
COPD: chronic obstructive pulmonary disease
COVID-19: coronavirus disease 2019
CRP: C-reactive protein
CT: computed tomograms
DIC: disseminated intravascular coagulation
ESR: erythrocyte sedimentation rate
GGO: ground-glass opacities
RT-PCR: reverse-transcription-polymerase-chain-reaction
SARS-CoV-2: severe acute respiratory syndrome coronavirus 2
SD: standard deviations
TSS: total severity score
WHO: World Health Organisation
